# Shoulder Instability Pediatric Management of Shoulder Instability in the Pediatric Athlete: A Contemporary Review of Current Evidence

**DOI:** 10.1007/s12178-026-10062-7

**Published:** 2026-10-21

**Authors:** Liam H. Wong, Hannah M. Chi, Erick M. Marigi, Nirav K. Pandya, Michael R. Davies

**Affiliations:** 1 Department of Orthopaedic Surgery, University of California, San Fracisco, San Francisco, CA USA; 2 Department of Orthopedic Surgery, Mayo Clinic Florida, Jacksonville, FL USA; 3 Department of Orthopaedic Surgery, University of California San Francisco, 1500 Owens St, San Francisco, CL 94158 USA

**Keywords:** Shoulder Instability, Pediatric Shoulder, Pediatric Shoulder Dislocation, Shoulder Instability Management

## Abstract

**Purpose of Review:**

Shoulder instability in pediatric and adolescent athletes is increasingly common and clinically complex due to early sport specialization and high-demand participation. This review aims to inform clinical decision-making by synthesizing contemporary evidence on injury patterns, diagnostic techniques, treatment options, and associated outcomes when faced with treating pediatric athletes.

**Recent Findings:**

Unique anatomic and developmental features specific to the pediatric population including capsular elasticity, collagen composition, open physes, and evolving glenoid and humeral morphology contribute to age-specific injury patterns and influence treatment outcomes. Younger children more frequently sustain atypical soft-tissue lesions, while adolescents experience markedly higher recurrence risk with rates of 44–77% following nonoperative management. Adolescents also develop off-track Hill-Sachs lesions more frequently than adults (24.4% vs. 3.3%), reflecting differences in immature bone mechanics. Diagnosis requires thorough history, physical examination, assessment of social and socioeconomic factors, and advanced imaging. Arthroscopic Bankart repair remains the most common surgical intervention and yields high return to sport rates (75–90%), though recurrence in pediatric patients (15–50%) substantially exceeds adult rates. Remplissage reduces recurrence in adolescents with off-track lesions, and bone augmentation procedures such as the Latarjet demonstrate lower recurrence (3–8%) in patients with significant glenoid bone loss or failed prior stabilization. Long-term outcomes in skeletally immature patients and alternative bone block procedures remain incompletely studied.

**Summary:**

Management requires individualized risk stratification based on adherence, skeletal maturity, sport demands, bone loss, lesion morphology and patient history and adherence. Although surgical stabilization provides favorable functional outcomes and high return to sport rates, recurrence remains a significant concern in younger and collision-sport athletes.

## Introduction

Shoulder instability in pediatric and adolescent patients is an increasingly prevalent and challenging problem [1, 2]. This increase is likely driven by early sport specialization, year-round training, and high-intensity athletic participation [3]. As such traumatic glenohumeral instability is now encountered more frequency in adolescents, particularly males involved in contact and overhead sports [2, 4]. Instability can be associated with substantial morbidity related to pain, functional limitation, and recurrent instability episodes [1, 2]. In the United States, the incidence of pediatric shoulder dislocation is approximately 60.3 per 100,000 patients younger than 18 years, with the highest rates in 16-year-old males (164.4 per 100,000 person-years) [4, 5]. Appropriate management of shoulder instability in skeletally immature patients must account for unique anatomic, biomechanical, and developmental factors that influence injury patterns and treatment outcomes [1, 3].

The pediatric shoulder differs fundamentally from the adult shoulder in its soft-tissue composition, bone morphology, and growth potential [1, 6]. Younger children with open physes generally demonstrate lower re-dislocation rates, whereas adolescents nearing skeletal maturity experience recurrence rates comparable to young adults, particularly following nonoperative management [2, 7, 8]. Primary anterior shoulder dislocation carries a high risk of recurrence, ranging from 44% to 77% with nonoperative treatment [2, 9]. Although surgical stabilization significantly reduces recurrence compared with nonoperative care (18% vs. 71%), recurrent instability after arthroscopic stabilization has been reported in up to 50% of pediatric patients [10, 11]. Therefore, the optimal treatment of shoulder instability, particularly following a first-time dislocation remains controversial. This review aims to inform clinical decision-making by synthesizing contemporary evidence on injury patterns, diagnostic techniques, treatment options, and associated outcomes when faced with treating pediatric athletes.

## Anatomy and Developmental Considerations

The glenohumeral joint relies on a balance of static and dynamic stabilizers to maintain stability through a wide range of motion. It consists of a large humeral head articulating with a relatively small, shallow glenoid fossa. The fibrocartilaginous labrum deepens the socket and centers the humeral head while static stabilizers (glenoid, labrum, capsule, glenohumeral ligaments) provide direction-specific restraint [12–14]. In the pediatric athlete, the inferior glenohumeral ligament (IGHL) serves as the principal passive stabilizer providing restraint to anterior and inferior translation at higher abduction (90°) and external rotation [13, 15, 16]. Dynamic stability is primarily provided by the rotator cuff muscles, which compress the humeral head into the glenoid. The long head of the biceps tendon (LHBT) contributes to additional anterior and superior stability, particularly during overhead activity [17, 18]. The supraspinatus, subscapularis, LHBT, along with the coracoacromial arch and acromioclavicular joint serve as additional passive stabilizers to superior humeral head migration during arm elevation and forceful overhead actions [12, 17].

The anatomy of the pediatric shoulder differs from the adult shoulder in ways that have important clinical implications for instability patterns and recurrence risk. Children and younger adolescents have increased capsular elasticity and a higher proportion of type III collagen, resulting in greater compliance but reduced resistance to repetitive loading and instability [7, 19, 20]. The pediatric shoulder capsule also contains a greater density of elastic fibers compared to adults, which theoretically helps absorb initial dislocation forces but paradoxically predisposes the joint to recurrent subluxation with the underlying elasticity [7, 19, 20]. With skeletal maturity, collagen composition shifts toward type I collagen, increasing capsular stiffness and susceptibility to permanent deformation following traumatic injury [21, 22].

Regarding bone mechanical behavior, children’s cortical bone has been shown to have 34% lower compressive Young’s modulus, 38% lower yield stress, and 33% lower ultimate stress compared to adult bone tissue [23]. In practical terms for shoulder injuries, this means that when a child sustains a traumatic shoulder dislocation, the softer humeral head bone (lower Young’s modulus) compresses more easily against the glenoid rim, and the lower yield and ultimate stress means increased likelihood of deformity at lower impact forces.

The proximal humeral physis also plays an important role in pediatric shoulder instability. Although largely extraarticular, the joint capsule inserts on the proximal humerus at an angle of approximately 14.5° relative to the anatomic neck, placing portions of the physis in close proximity to the capsule [24]. Consequently, physeal injury may coexist with instability events [1, 25, 26]. Recognition of open physes is essential when evaluating imaging, determining surgical indications, and counseling families regarding prognosis. Importantly, children with an open proximal humeral physis are significantly less likely to experience recurrent instability compared with skeletally mature adolescences. Deitch et al. reported that adolescents with closed physes were 14 times more likely to develop recurrent instability than those with an open physis [8]. This protective effect likely reflects the greater tissue compliance and energy-absorbing capacity of the immature skeleton which will deform and partially remodel following a dislocation event without sustaining the permanent capsuloligamentous injury that increases risk of recurrence [1, 7]. As the physis closes and capsular collagen matures to type I collagen, the glenohumeral joint progressively loses that protective laxity [7, 20]. During this time, the glenoid fossa deepens with ossification of the secondary ossification center and remodels in response to loading [27, 28]. The humerus also decreases in retroversion through adolescence [29, 30]. These developmental changes influence both injury patterns and the biomechanical rationale for timing and indications for specific surgical interventions.

## Pathogenesis

Pediatric shoulder instability exists along a spectrum ranging from acute traumatic dislocation to atraumatic multidirectional instability (MDI). Traumatic anterior instability is the most common pattern occurring when an anteriorly directed force is applied to the abducted, externally rotated shoulder, resulting in injury to the anteroinferior capsulolabral complex [1, 2]. While Bankart lesions (anteroinferior labral injury) and Hill-Sachs lesions (HSLs) (posterosuperior humeral head impaction fractures from the glenoid rim) remain the most frequently encountered findings, their prevalence and morphology vary with skeletal maturity [1, 7, 31, 32].

Bony Bankart lesions are more common in adolescents aged 15 and older (34.2% versus 18.2%), whereas younger children under 15 years more frequently sustain atypical soft-tissue injuries [1, 19]. Anterior labrum periosteal sleeve avulsion (ALPSA) lesions occur in 23.6% of patients under 15 years compared to 10.5% in those 15 years and older [19]. When all atypical lesions (including ALPSA, humeral avulsion of glenohumeral ligament [HAGL] lesions, and subscapularis avulsions) are considered together, they are present in 41.8% of patients under 15 years versus only 17.1% in older adolescents [19]. These atypical lesions reflect the relative compliance of immature periosteal tissue, which tends to strip rather than tear [1, 19]. Shearing of the articular cartilage along with the labrum (glenolabral articular disruption [GLAD] injuries), axillary neuropraxia, rotator cuff tears, and superior labrum anterior-to-posterior (SLAP) lesions may also occur, but are associated with higher-energy mechanisms or recurrent instability [1, 7, 31–33].

The zone of humeral head contact during shoulder motion, the glenoid track, is especially relevant in the adolescent population. Calculated as 83% of the intact glenoid width (0.83D) minus the width of glenoid bone loss (d) (Glenoid Track = 0.83D – d) [34, 35]. HSLs confined within the glenoid track are considered on-track and are unlikely to engage the glenoid and lead to instability, whereas off-track lesions extend medial to the glenoid track and carry a high-risk of engagement and recurrent instability [34, 35]. Recurrent instability may lead to progressive glenoid bone loss and enlargement of HSLs, resulting in bipolar bone loss and further destabilization [31, 32]. Emerging evidence suggests that adolescents may develop off-track HSLs more frequently than adults (24.4% in adolescents compared with 3.3% in adults) [7, 36]. This predominance seen in adolescents is thought to be largely due to the combined effect of softer humeral bone (lower Young’s modulus) and increased propensity for deformity at the time of injury (lower yield and ultimate stress), and higher-energy mechanisms common in collision sports, producing larger humeral head defects with lower impact forces relative to glenoid dimensions [1, 19, 23].

Traumatic unidirectional instability accounts for the majority of pediatric instability presentations, with anterior instability representing the dominant subtype in pediatric and adolescent athletes [4, 5]. In contrast, MDI is comparatively less common but is proportionally overrepresented in younger children, although no studies have reported universal incidence [1]. MDI is typically associated with generalized ligamentous laxity, connective tissue disorders (e.g., Ehlers–Danlos), and deficits in neuromuscular control [1, 31]. MDI spans a continuum from recurrent subluxation to frank dislocation in multiple directions; it often presents with instability during mid-range motion, reflecting dynamic stabilizer dysfunction rather than discrete structural injury [1, 31, 37]. Muscle abnormalities and scapular dyskinesis additionally play a central role in MDI pathogenesis and treatment expectations [1, 31, 37].

## Diagnosis

### History and Physical Examination

A detailed history and physical examination are critical for differentiating instability subtypes and guiding management the pediatric population. Key historical elements include age at first instability event, mechanism of injury, number of prior dislocations or subluxations, need for reduction in the emergency department, hand dominance, sport participation (collision versus overhead), season timing, and prior treatment, including adherence and response to physical therapy [38]. Family history and systemic features suggestive of connective tissue disorder (skin findings, dystrophic scars, striae) and cardiovascular red flags (elevated blood pressure for age, heart murmurs, irregular rhythm, poor exercise response) should also be assessed [39].

Traumatic instability is typically described as a sudden “pop” accompanied by acute pain and weakness and may include transient axillary nerve paresthesia. Atraumatic instability often manifests as recurrent nonpainful subluxations or self-reducing dislocations. Most shoulder dislocations spontaneously reduce on the field; however, fracture must be excluded before attempted reduction by a trained provider [1].

Physical examination begins with inspection and comparison of range of motion, strength, and neurovascular status with the contralateral unaffected shoulder, with particular attention to axillary nerve function [38, 40]. Several provocative tests are then performed. Anterior instability is suggested by apprehension in 90° abduction and external rotation (apprehension test) and confirmed by relief with posterior pressure (relocation test) [41]. The apprehension test demonstrates a sensitivity of 72% and specificity of 96% when used as the diagnostic endpoint [42]. Glenohumeral translation is assessed with the load-and-shift test and graded as I (increased translation without subluxation), II (subluxation where the humeral head translates to the glenoid rim but not over it), or III (dislocation over the glenoid rim) [41] Posterior instability and MDI are evaluated similar to adults using the posterior apprehension test, jerk test, Kim test, and sulcus sign [43]. Assessment for generalized hypermobility using the Beighton score is also recommended, with scores ≥ 4 suggesting hypermobility [44].

Insurance status is an important socioeconomic factor to recognize during diagnosis that can meaningfully influence both the severity of pathology at presentation and surgical outcomes in pediatric shoulder instability. Hung et al. found that publicly insured pediatric patients experienced significantly longer delays to evaluation and advanced imaging, were twice as likely to have developed secondary bony injuries and had a significantly higher rate of postoperative re-dislocation compared with privately insured pediatric patients (24.3% versus 0.0%) [45].

### Imaging and Diagnostic Studies

Initial imaging among pediatric and adolescent patients should include a true anteroposterior (AP) (Grashey view), scapular Y, and axillary views to confirm reduction and assess for fractures. Obtain at least two orthogonal views to avoid missing posterior dislocations [38]. A Velpeau view may be used when an axillary view is not tolerated. Specialized views include the West Point view for glenoid bone loss and Bankart lesions or the Stryker notch view for HSLs. Traumatic dislocations can be associated with greater or lesser tuberosity fractures; however, open physes in the shoulder, such as the coracoid base and glenoid physes, should not be misinterpreted as fractures in skeletally immature patients [46].

Magnetic resonance imaging (MRI) is generally indicated in this population to evaluate associated soft-tissue pathology, cartilage injury, marrow edema, and quantify the degree of glenoid bone loss [1, 3, 31]. MRI can characterize degree of labral pathology such as Bankart lesions with or without SLAP lesions, rotator cuff injury, as well as the presence and type of HSL (on-track versus off-track) [1, 34, 35]. MRI can characterize other lesions including ALPSA, HAGL, and GLAD lesions [1, 7, 31, 32]. Although, the routine MRI after first-time dislocation in pediatric patients remains controversial, there is an increase in use and indication with high impact sports and mechanism of dislocation [3].

Computed tomography (CT) is typically reserved for patients with recurrent instability or preoperative planning to quantify bone loss [1, 25]. CT with 3D reconstruction allows precise glenoid bone loss measurement using the best-fit circle method on an enface glenoid fossa view [35, 47]. This method quantifies the glenoid bone loss by calculating the ratio of defect width to the diameter of a best-fit circle of the expected inferior two-thirds of the native glenoid [35, 47]. Important for the pediatric population, MRI has demonstrated comparable accuracy to assessing glenoid bone loss to 3D CT scans while avoiding radiation exposure [48, 49].

### Differential Diagnosis

Providers should maintain a thorough differential diagnosis in pediatric patients experiencing symptoms related to shoulder instability. While many pediatric athletes do experience shoulder instability from a traumatic event, other diagnoses to maintain include MDI, impingement, Little League shoulder, acromioclavicular joint separation, shoulder contusion, scapular dyskinesis, distal clavicle osteolysis, SLAP tear, proximal humerus fracture, clavicle fracture, and biceps tendonitis [50].

## Nonoperative Management

Initial management for pediatric and adolescent athletes typically includes immobilization, pain control, and early rehabilitation emphasizing restoration of motion, rotator cuff strength, and scapular control. In-season management may prioritize expedited functional recovery with explicit counseling about recurrence risk. Some protocols describe 4–5 weeks of focused physical therapy before sport-specific progression if the athlete regains full motion/strength and lacks apprehension.

The optimal method and duration of immobilization after closed reduction remain controversial. Traditional immobilization is performed with the shoulder in internal rotation, although Itoi et al. reported a reduced recurrence risk with external rotation immobilization in patients younger than 30 years with first-time anterior dislocations [51]. Subsequent studies failed to confirm a difference in recurrence between internal and external rotation immobilization [52, 53], and no consensus exists regarding duration, with protocols ranging from less than one week to over six weeks. A meta-analysis by Paterson et al. found no difference in recurrence between immobilization for one week or less versus three weeks or more in patients under 30 years [53]. At our institution, short-term sling immobilization (7–10 days) followed by progressive rehabilitation is commonly employed.

Recurrent instability rates in adolescents after nonoperative management remain high, ranging from 56% to over 80% [54, 55]. In a cohort of 133 adolescents with primary anterior dislocation, Roberts et al. reported a recurrence rate of 76.7%, with a median time to re-dislocation of 10 months [9]. Risk factors for recurrence include male sex, skeletal immaturity, participation in high-impact or collision sports, ligamentous laxity, and the presence of Bankart lesions, HSLs, or glenoid bone loss [7, 55–57].

Despite the high recurrence rate, nonoperative management is often chosen to allow in-season return to play, provided athletes regain painless, symmetric range of motion and functional strength. Return to sport may occur as early as two weeks in select cases [58], although protective bracing has not been shown to reliably prevent recurrence [59]. Given high recurrent instability risk, patients and families should be counseled regarding the likelihood of needing surgical intervention, particularly in the off-season.

Patients with atraumatic multidirectional instability should be primarily managed nonoperatively [60]. Many patients with MDI achieve excellent outcomes with nonoperative treatment focused on neuromuscular control and scapulothoracic stabilization [61].

## Case Example

**Fig. 1:**
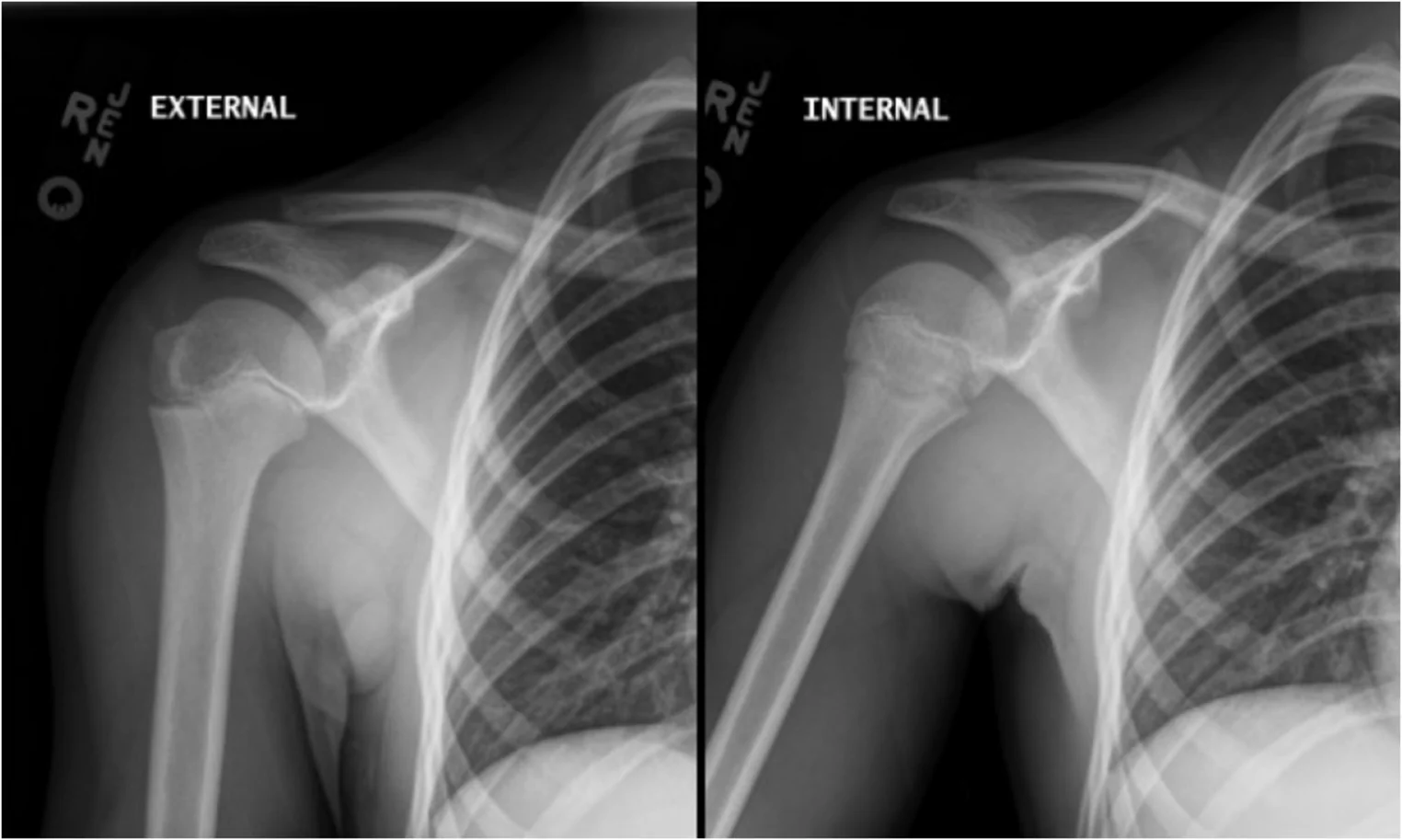
Anterior-posterior XR of right shoulder demonstrating skeletally immature shoulder

**Fig. 2:**
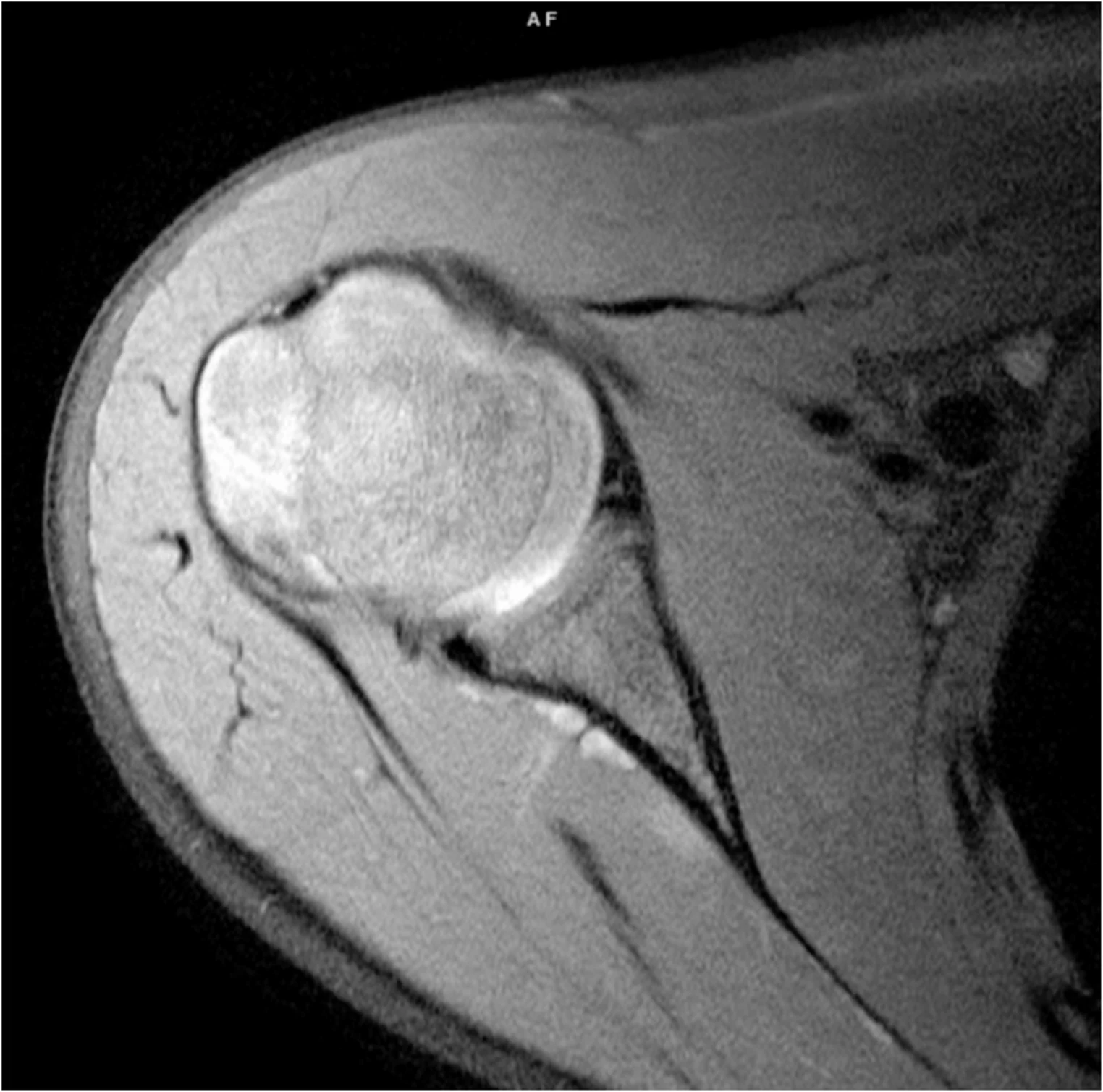
Axial shoulder magnetic resonance imaging demonstrating anteroinferior labral tear

The first patient is a 13-year-old male who presented with several years of recurrent shoulder dislocations after initial injury occurred at age seven after a fall onto the right shoulder. The patient trialed nonoperative management before they presented for surgical management. The patient endorsed multiple experiences of a “pop” and would need to manually self-reduce the shoulder. He participated in soccer, football, and basketball; however, he wanted to return to boxing. Physical exam demonstrated full active range-of-motion with normal strength. The patient had positive apprehension and relocation test, as well as positive O’Brien test and signs for impingement. Preoperative imaging demonstrated a skeletally immature shoulder (Fig. 1). MRI demonstrated an anteroinferior labrum tear without a HSL (Fig. 2).

**Fig. 3:**
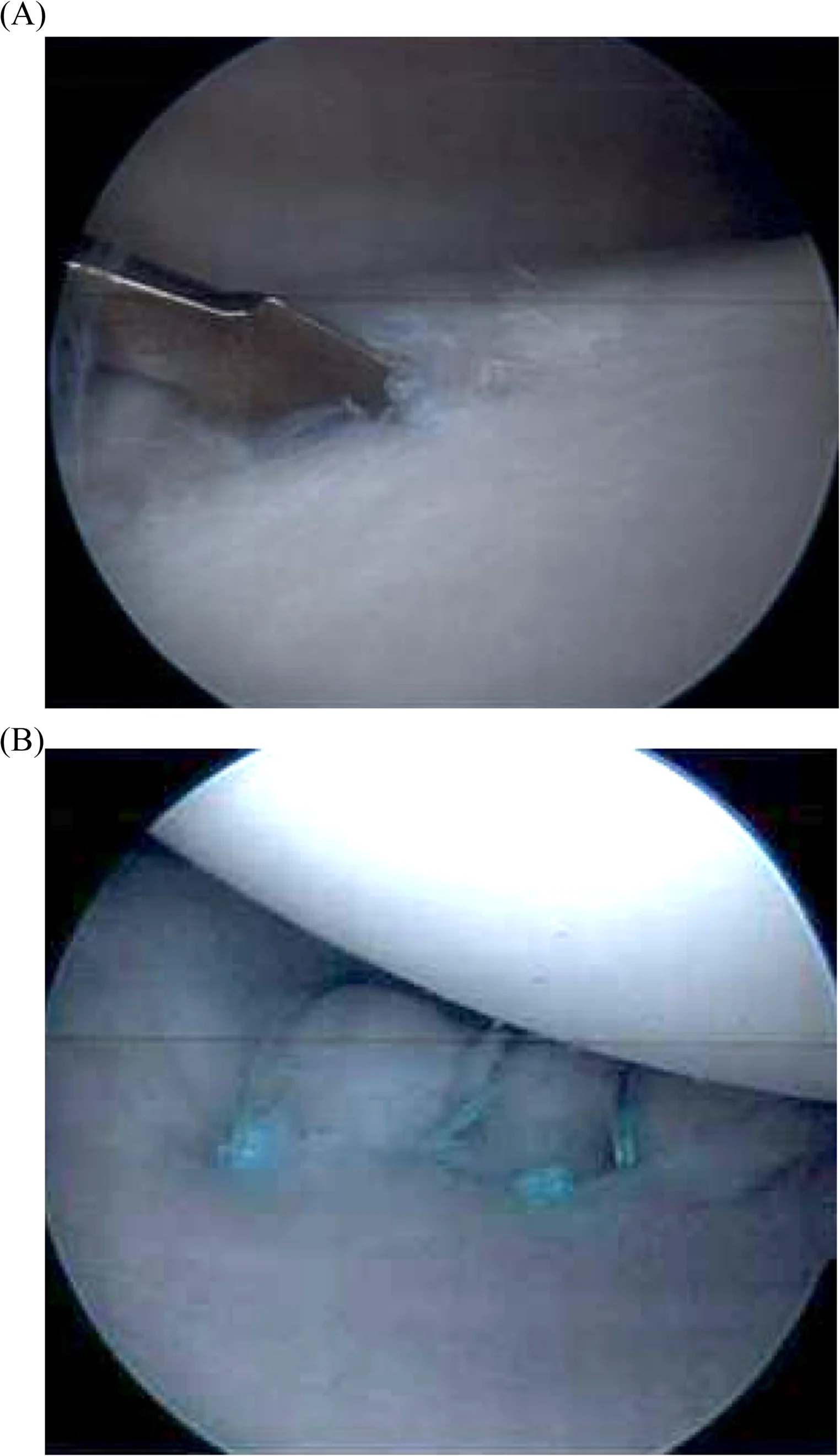
Beach chair position arthroscopic debridement of an anteroinferior labral tear before (**A**) and after (**B**) labral repair

Initial diagnostic arthroscopy confirmed a soft tissue Bankart lesion (2:30 − 6:30) without other lesions or pathology. The procedure continued with arthroscopic Bankart repair starting with a rasp to mobilize the labrum and create a bony bed for healing (Fig. 3A). Mobility of the labrum and ability to shift back to its anatomic location was confirmed. Final repair was performed with three knotless suture anchors starting most inferiorly grabbing capsulolabral tissue at 6:30 and advancing to 5:00 and continuing up the glenoid (Fig. 3B).

**Fig. 4:**
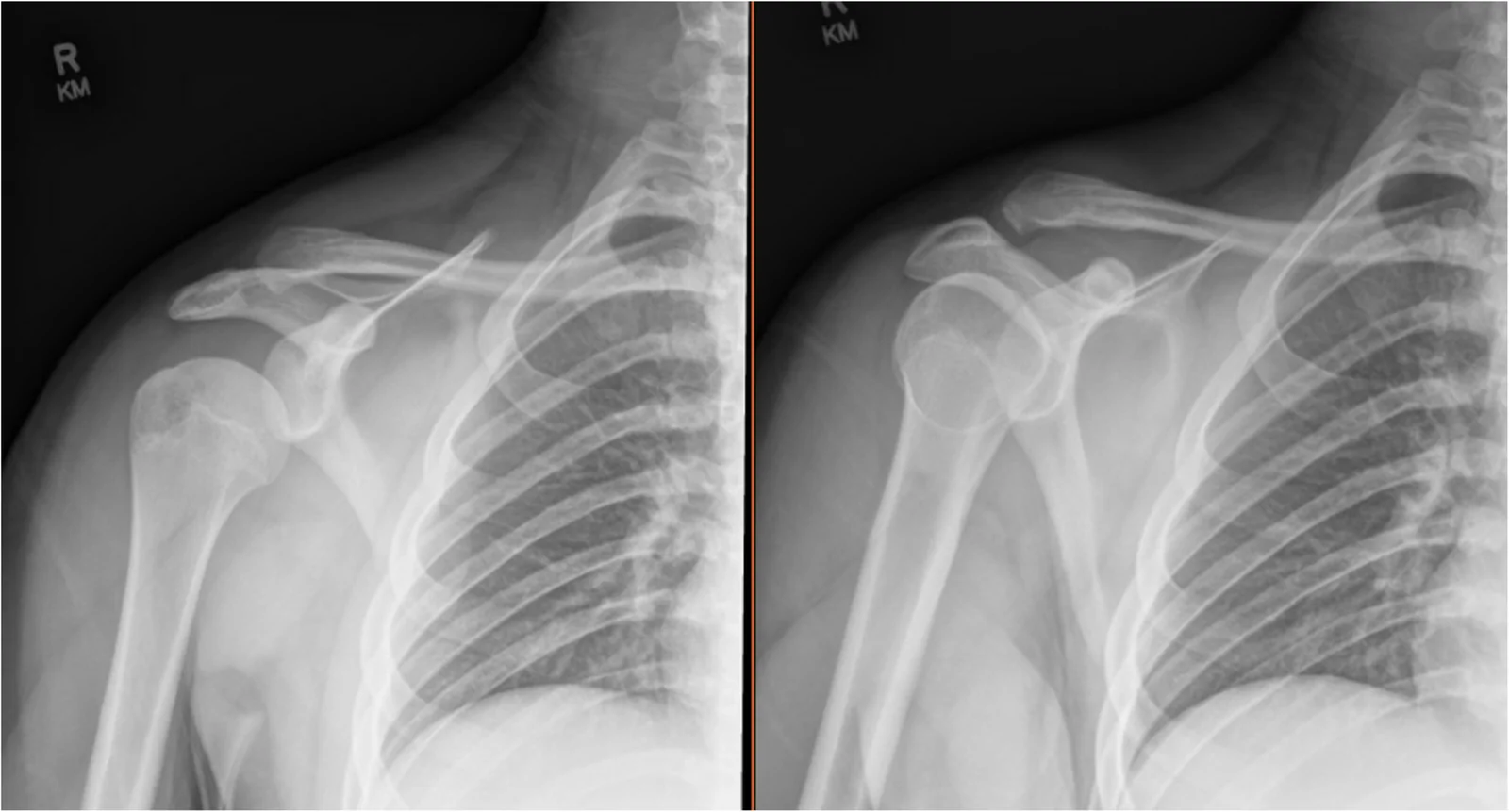
Anterior-posterior XR of right shoulder demonstrating skeletally mature shoulder with inferior subluxation

**Fig. 5:**
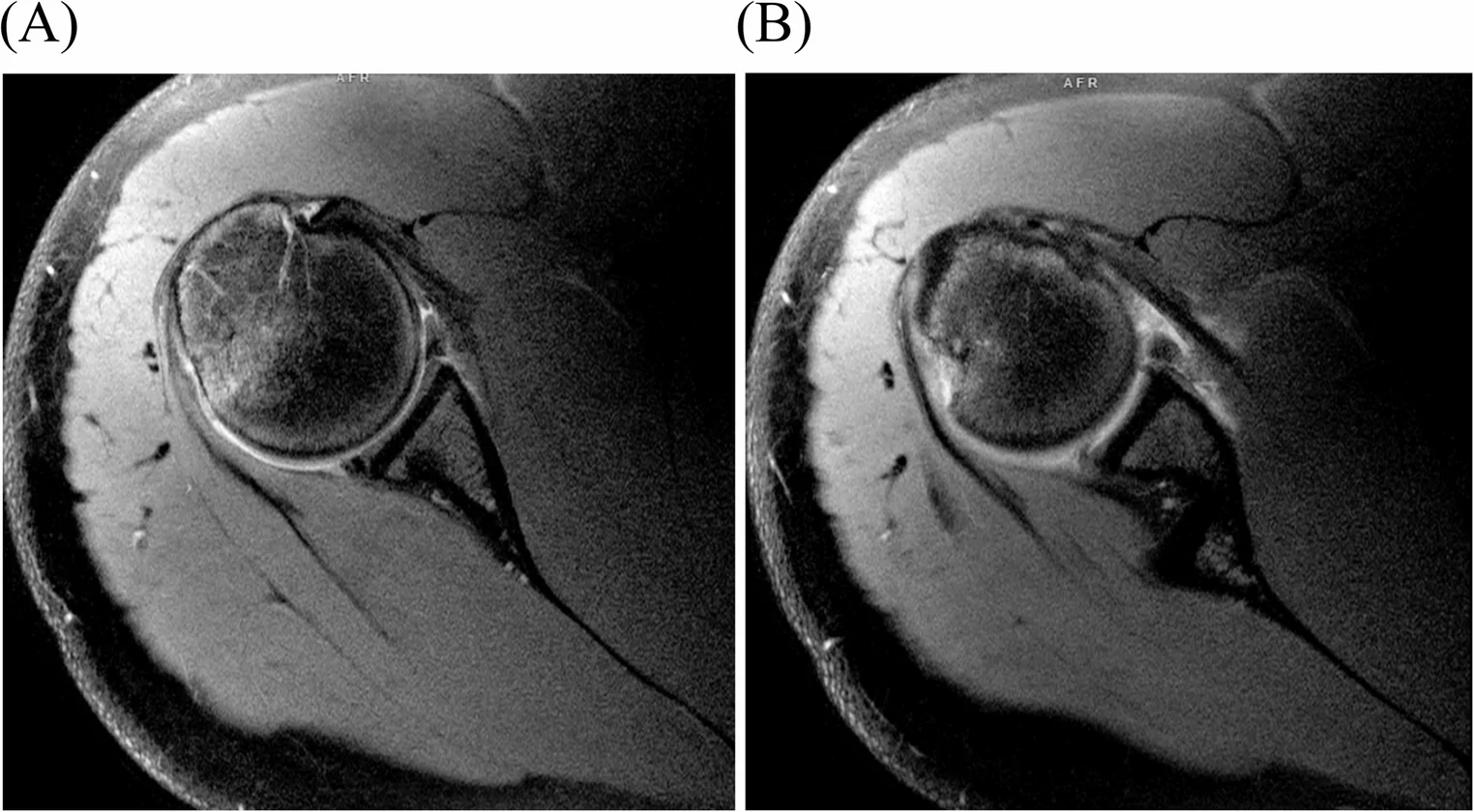
Axial shoulder magnetic resonance imaging demonstrating (**A**) anteroinferior labral tear and (**B**) Hill-Sachs lesion

The second patient is a 16-year-old male who presented with one year of recurrent shoulder instability after initial shoulder dislocation during football practice. The patient endorsed numbness and tingling at time of first injury that has since improved. Physical exam demonstrated full active range of motion with normal strength. The patient had positive apprehension and relocation test, as well as positive O’Brien test and signs for impingement. Preoperative imaging demonstrated a skeletally mature shoulder with inferior subluxation (Fig. 4). MRI demonstrated an anteroinferior labrum tear (Fig. 5A) with a Hill-Sachs lesion (Fig. 5B).

**Fig. 6:**
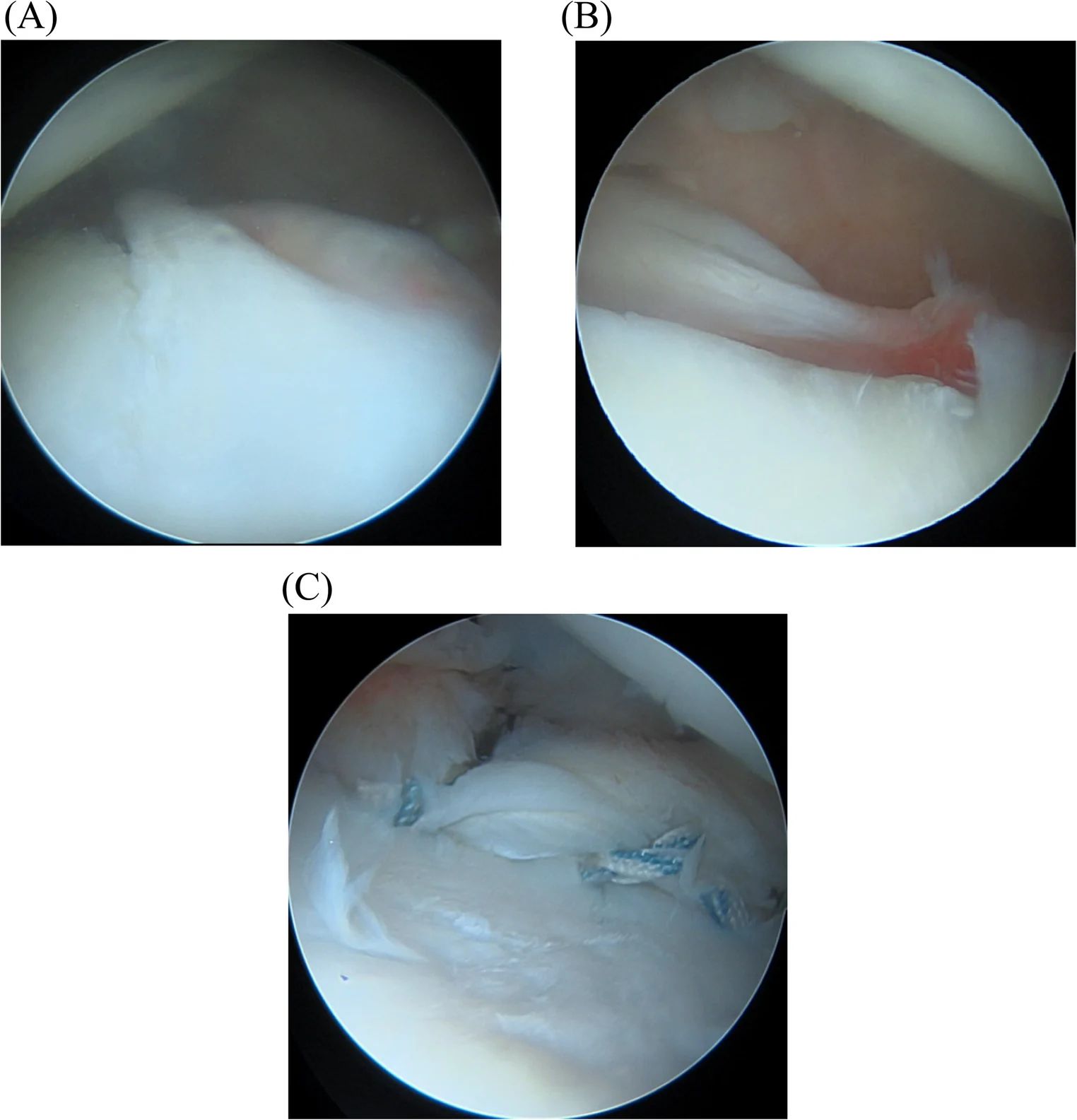
Beach chair position arthroscopic demonstration of Hill-Sachs lesion (**A**), and debridement of an anteroinferior labral tear before (**B**) and after (**C**) labral repair

Initial diagnostic arthroscopy confirmed a soft tissue Bankart lesion (2:00–7:00) as well as a non-engaging on-track HSL (Fig. 4A). The procedure continued with arthroscopic Bankart repair starting with a rasp to mobilize the labrum and create a bony bed for healing (Fig. 4A). Mobility of the labrum and ability to shift back to its anatomic location was confirmed. Final repair was performed with four knotless suture anchors starting most inferiorly at the 5:00 position, 4:00, 3:00, and 1:30 position taking care to advance the labrum and capsule anteriorly-superiorly (Fig. 4B). Given the small size and on-track HSL, no remplissage was performed Fig. 6.

## Surgical Management

### Indications and Procedure Selection

Indications for surgical management of anterior shoulder dislocation in pediatric patients remain controversial, particularly after a first-time event. Although nonoperative treatment has traditionally been favored, growing evidence demonstrates high recurrence rates with conservative management, especially in skeletally immature patients and those participating in high-demand or collision sports [54–57]. Consequently, early surgical stabilization is increasingly considered in select high-risk pediatric patients. Stabilization after a first-time dislocation in pediatric patients remains controversial but literature supports a risk-stratified approach [3]. A meta-analysis by Zaremski et al. demonstrated that primary operative treatment was associated with significantly lower recurrence rates compared with nonoperative management in youth athletes aged 14 years and older (OR 13.41), with return to sport rates of 95.3% operative versus 41.3% nonoperative [36]. However, athletes younger than 14 years in the nonoperative group were less likely to recur compared with those aged 14 years and older. This suggests that early primary stabilization may be most beneficial in older adolescents approaching skeletal maturity.

Surgical intervention should be considered in high-risk patients as recurrent instability is common and may contribute to progressive glenoid and humeral head bone loss [2, 7]. Key indications for early surgical stabilization after a first-time dislocation include off-season timing, presence of a bony Bankart lesion or ALPSA lesion, bipolar bone loss (including an off-track HSL), or a high-demand overhead or collision-sport athlete unwilling to accept recurrence risk [3]. Patients with an open proximal humeral growth plate, those who are in-season, or those averse to surgery may reasonably pursue an accelerated rehabilitation program with understanding that recurrence risk remains substantial. Additionally in the pediatric and adolescent athlete population, reviewing and carefully assessing the likelihood of postoperative adherence with activity restrictions and rehabilitation protocols is a critical preoperative consideration, as younger patients and their families may struggle to adhere to the prolonged immobilization and physical therapy demands that are essential for a successful outcome. Surgeons should engage both the patient and their families in detailed counseling prior to proceeding with stabilization to ensure realistic expectations and commitment to the postoperative course.

Surgical stabilization is more clearly indicated in pediatric patients with recurrent anterior instability who have failed appropriate nonoperative management, including immobilization and structured rehabilitation [62]. Recurrent dislocations or subluxations that impair function, limit sports participation, or occur with progressively lower-energy mechanisms are strong indications for operative reconstruction. In contrast, surgical management should be approached cautiously in pediatric patients with MDI, voluntary dislocations or subluxations, chronic pain without discrete instability events, and those with significant medical or psychosocial comorbidities, as outcomes are less predictable [31]. Careful patient selection and thorough preoperative evaluation remain essential to optimizing outcomes in pediatric shoulder instability.

### Diagnostic Arthroscopy

After diagnosis and recommendation for surgical management, diagnostic arthroscopy is performed in all cases to confirm pathology and determine the need for adjunct procedures. Intra-articular structures influencing recurrence risk including the rotator cuff, biceps, superior and posterior labrum, and capsule / labrum should be systematically assessed. Discrepancies from preoperative MRI or interval changes should be documented. The extent of the anterior labral tear, including tissue quality and any associated bony involvement, can be confirmed.

Our institution highlights several technical considerations and key intraoperative findings important for the pediatric and adolescent population. Posterior-superior labral peel-back indicating a SLAP lesion is best assessed with the arm in abduction and external rotation. Traction time should be minimized (< 1 h) to prevent neurovascular injury. A drive-through sign, defined by easy passage of the arthroscope between the humeral head and glenoid at the level of the IGHL, indicates significant capsular laxity and is commonly observed in MDI.

### Arthroscopic Bankart Repair

Arthroscopic Bankart repair is widely used in pediatric athletes and typically yields high return to sport rates, although recurrence remains substantially higher than in adults. Across pediatric and adolescent cohorts, recurrent instability rates range from approximately 15% to over 50%, influenced by age, sport participation, and underlying structural pathology (Table 1) [32, 63–66]. A systematic review by Warner et al. reported a recurrence rate of 26.7% (203/761 shoulders), with 23.1% (76/329 shoulders) requiring revision surgery [10]. These rates exceed those reported in adult populations (8.1–14.8%) [16, 67, 68], particularly among younger adolescents and collision-sport athletes [65].

**Table 1:** Rate and predictors of recurrent instability following arthroscopic Bankart repair in pediatric and adolescent patients

Study	Arthroscopic Bankart Repair Population	Recurrent Instability Rate	Risk Factors for Recurrent Instability
Egger et al. 2023 [64]	59 patients, median age 16 years (range 12–18)	At a mean of 49 months follow-up: Recurrent instability 18/59 (31%) Revision stabilization 10/59 (17%) Subjective instability 8/59 (14%)	Tear length and number of anchors on univariate analysis; larger Hill-Sachs lesions on MRI associated with revision surgery
Cheng et al. 2021 [32]	171 patients, mean age 15.9 years	At a mean 5.4 years follow-up: Recurrent instability 35/171 (21%)	More than one dislocation, open physis, glenoid retroversion < 6°
Waltenspul et al. 2022 [65]	35 shoulders, mean age 16.4 years (range 12–18)	At a mean of 4 years follow-up: Recurrent instability 20/35 (57%) Revision stabilization 13/35 (37%)	Younger age at the time of the surgical procedure
Torrance et al. 2018 [66]	67 patients, mean age 16.3 years (range 14–17), participating in collision sports	At a mean of 33 months follow-up: Recurrent instability 34/67 (51%) Revision stabilization 30/67 (45%)	Age < 16 (versus those ≥ 16 years)
Nakagawa et al. 2023 [67]	115 patients, mean age 17 years (standard deviation 2 years)	At a mean of 30 months follow-up: Recurrent instability 16/115 (14%)	Preoperative glenoid defect size and bone union on univariate analysis

Risk factors for recurrent instability after arthroscopic Bankart repair consistently include younger age, greater glenoid bone loss, and the presence and size of a HSL [10, 32, 63–66]. Additional predictors specific to pediatric and adolescent patients include multiple preoperative dislocations, participation in overhead or collision sports, and larger labral tears [10, 32, 63–66]. Younger age at surgery is one of the most reproducible risk factors, with recurrence rates exceeding 50% in patients younger than 16 years in some series [64, 65]. A recent systematic review further demonstrated higher recurrence in patients younger than 20 years compared with older cohorts (29.1% [101/346] vs. 13.2% [100/755]) [69]. Bony morphology and bipolar bone loss additionally play an important role in failure after arthroscopic Bankart repair in pediatric athletes. Larger preoperative glenoid defects and greater HSL size on MRI have been independently associated with recurrent instability and revision surgery, emphasizing that even subcritical bone loss may be clinically relevant in young, high-demand patients [63, 66].

Despite the elevated risk of recurrent instability, arthroscopic Bankart repair generally yields favorable functional outcomes and high return to sport rates in pediatric athletes. Reported return to sport rates range from 75% to over 90%, with most athletes competitively returning within 5–7 months postoperatively [70–72]. In a prospective series of 67 young athletes (mean age 17.5 years), 88% returned to sport at an average of 7.1 months, with 75% returning to the same level or higher [71]. However, return to preinjury level varies by sport type, particularly in collision and overhead sports [70–73]. In one study of teenage athletes, 76% achieved complete return to preinjury level without complaints, but varied significantly by sport type: 82% in contact athletes, 59% in dominant-hand overhead athletes, and 100% in other athletes [70].

Patient-reported outcome measures (PROMs) including the American Shoulder and Elbow Surgeons (ASES) score, Rowe score, Patient Acceptable Symptom State (PASS), and Single Assessment Numeric Evaluation (SANE) score typically show significant improvements [63, 74, 75]. PROMs may decrease in patients who experience recurrent instability or subjective apprehension, even in the absence of frank re-dislocation [63, 74]. Notably, subjective instability without complete dislocation represents a clinically meaningful failure mode in pediatric athletes and may negatively affect confidence and performance in high-demand athletes [63].

Aside from recurrent instability, complications following arthroscopic Bankart repair in pediatric athletes are relatively uncommon. Revision stabilization rates range from approximately 10% to 35%, with higher rates again in younger patients and collision-sport athletes [10, 31, 63, 71, 76, 77]. A large cohort study of 488 adolescents demonstrated cumulative revision rates of 8.8% at 2 years, 16.5% at 5 years, and 20% at 15 years [31]. Revision procedures often require more complex strategies, such as remplissage or bone augmentation [10]. Less common complications include postoperative stiffness, persistent pain, and anchor-related issues [74, 77, 78]. Loss of external rotation is uncommon but may become clinically relevant in overhead athletes, particularly if an aggressive capsular shift is performed [77].

Several key technical considerations from our institution are highlighted. Surgical success is supported by thorough liberation and mobilization of the labrum, preparation of bleeding bone along the glenoid rim, and advancement of both the labrum and capsule. Placement of at least three anchors, starting as inferiorly as possible with appropriate spacing, is recommended, while avoiding anchor placement near the biceps anchor to prevent constraint of the superior labrum.

### Remplissage

Remplissage is increasingly used as an adjunct to arthroscopic Bankart repair in pediatric athletes, particularly those with off-track HSLs. The fixation of the posterior capsule and infraspinatus tendon to the humeral head defect enhances resistance to anterior translation, converting the HSL to an extra-articular defect [10]. Pediatric comparative data is limited; however, Hughes et al. reported lower recurrent instability with remplissage compared with Bankart repair alone (13% [2/15] versus 47% [8/17]) in adolescents, with no significant differences in patient-reported outcomes [79]. Although postoperative loss of external rotation has not been clearly demonstrated in pediatric cohorts, adult studies (particularly in throwing athletes) have reported this risk [80, 81]. Notably, excessive medial placement of remplissage anchors has been associated with greater external rotation loss and glenohumeral cartilage degeneration in adult patients, emphasizing the importance of proper anchor positioning within the Hill-Sachs defect [82, 83].

At our institution, remplissage is strongly considered for high-risk adolescents, including those with engaging or off-track HSLs, significant humeral bone loss, younger age, multiple preoperative dislocations, or participation in collision sports. Overhead and throwing athletes should be counseled regarding potential sport-specific limitations, particularly related to throwing mechanics.

### Bone Augmentation

Bone augmentation procedures (such as the Latarjet procedure) represent an important treatment option in pediatric and adolescent patients with substantial glenoid bone loss, high-risk instability profiles, or those who have failed arthroscopic stabilization. The Latarjet procedure improves stability by increasing glenoid width and providing a dynamic sling effect. In a cohort of 41 adolescents, Waltenspül et al. reported lower recurrence rates and improved PROMs following open Latarjet compared to arthroscopic Bankart repair [64]. Recurrent instability is generally lower than arthroscopic Bankart repair ranging 3% to 8% in recent studies (Table 2) [54, 64, 84–86]. Additional studies have demonstrated excellent return to sport rates, preserved range of motion, and favorable functional outcomes after both primary and revision Latarjet procedures in adolescent patients [84, 85].

**Table 2:** Rate of recurrent instability and other complications following bone augmentation in pediatric and adolescent patients

Study	Bone Augmentation Population	Recurrent Instability Rate	Other Reported Complications
Waltenspül et al. 2022 [65]	31 shoulders, mean age 16.7 years (range 14–18)	At a mean of 6 years follow-up: Recurrent instability 2/31 (6%)	Screw irritation 4/31 (13%) Coracoid block fracture 1/31 (3%) Axillary nerve palsy 1/31 (3%) Pain after heavy activity 1/31 (3%) Scapular dyskinesia 1/31 (3%)
Rossi et al. 2022 [85]	60 shoulder, mean age 16.3 years (range 15–20)	At a mean of 4.8 years follow-up: Recurrent instability 2/60 (3.3%)	Nonunion 4/60 (7%) Graft malposition 4/60 (7%) Infection 1/60 (2%) Hematoma 1/60 (2%) Mild osteoarthritis 1/60 (2%)
Rosello et al. 2024 [86]	24 shoulders, mean age 16.5 (range 13.5–18.0)	At a mean of 4.1 years follow-up: Recurrent instability 2/24 (8%)	Infection 1/24 (4%) Nonunion / migration 8/24 (33%) Osteolysis 8/24 (33%)
Delgado et al. 2024 [87]	17 shoulders, mean age 18 (range 15–19)	At a median of 9 years follow-up: Recurrent instability 1/18 (6%)	Screw irritation 1/17 (6%)
Khan et al. 2014 [54]	28 shoulders, mean age 15.9 years (standard deviation 4.3 years)	At a mean of 9.7 years follow-up: Recurrent instability 2/28 (7%)	Screw irritation 3/28 (11%)

Although a promising option after a failed Bankart repair and amongst adolescents with glenoid bone loss, meticulous attention to the smaller coracoid size and graft positioning is critical as complications may be particularly impactful in young patients. Complication rates after bone augmentation procedures have been reported as high as 30% in older patients [87]. In the adolescent cohort reported by Waltenspül et al., complications included two coracoid fractures and one transient axillary nerve palsy [64]. The safety and efficacy of the Latarjet procedure have been well studied in older populations, but studies amongst younger patients have been limited. While concerns persist regarding its use in skeletally immature patients, Domos et al. found no evidence of glenoid growth disturbance of deformity in 45 skeletally immature patients undergoing Latarjet stabilization [88]. The risk of postoperative osteoarthritis remains a major concern, with studies reporting up to a 25% incidence of moderate to severe osteoarthritis at 5 years and a 4.9 times increased risk when the graft overhangs the glenoid articular surface [89]. However, other studies suggest a lower osteoarthritis risk compared with arthroscopic Bankart repair or nonoperative treatment [90].

The Latarjet should be considered for adolescent patients with significant glenoid bone loss or have failed prior stabilization. While evidence suggests that the risk of complications remains appropriately low, care should be taken in considering a nonanatomic procedure in adolescent patients. Further research with larger cohorts and longer-term outcomes is necessary to better understand the nuanced indications and outcomes following bone augmentation procedures in adolescents. Additionally, there is a paucity of literature evaluating alternative bone block procedures, including distal tibial allograft (DTA) and iliac crest autograft reconstruction. DTA offers the theoretical advantage of an anatomically shaped, hyaline cartilage-bearing surface that better replicates native glenoid contour. In adults, DTA delivers comparable rates of instability and PROMs to Latarjet, with lower risk of complications [91]. However, no studies have evaluated DTA or iliac crest autograft reconstruction techniques in isolated pediatric or adolescent populations.

Several key technical considerations from our institution are emphasized with pediatric and adolescent patients. Neurovascular structures should be meticulously protected by minimizing medial dissection and remaining lateral when working inferior to the coracoid. Graft harvest should avoid excessive resection while ensuring adequate coracoid length. Accurate graft positioning is critical with screws placed parallel to the joint surface and without lateral prominence that should be confirmed with fluoroscopy. Intraoperative fluoroscopy is obtained angled roughly 30–45° from the coronal plane, perpendicular to the scapular plane, to produce a true AP (Grashey) view of the glenoid, confirmed when the anterior and posterior glenoid rims are superimposed and the joint space is clearly visualized, allowing assessment of graft position, screw trajectory, and the glenoid physis. Additionally, placement of the graft too lateral can lead to increased pain and potential for developing osteoarthritis whereas too medial can lead to persistent instability. When feasible, capsular repair over the graft can further enhance stability.

### Arthroscopic Capsular Plication

Patients with atraumatic multidirectional instability should be primarily managed nonoperatively [60]. When a traumatic component is present, further evaluation for associated labral pathology is warranted. In pediatric athletes who remain symptomatic despite prolonged rehabilitation, arthroscopic capsular plication may be indicated. The procedure addresses capsular redundancy by restoring capsular tension and improving stability without bony augmentation. Outcomes in pediatric patients are limited. In one study of 50 adolescent shoulders (mean age 15.8 years), 26% (13/50) experienced recurrent instability requiring reoperation and 56% returned to sport [92]. Recurrence rates are comparable to those observed after stabilization for traumatic unidirectional instability [76, 92]. Careful patient selection and counseling are essential, particularly in athletes with generalized ligamentous laxity or high athletic demands. Additionally, patients with higher Beighton scores of 4–9 are associated with smaller functional improvements and lower rates of excellent outcomes [93].

### Postoperative Care

Postoperative management following surgical stabilization for shoulder instability in the pediatric athlete is structured to protect the repair while allowing for gradual restoration of motion, strength, and function. Pediatric and adolescent patients are placed into a shoulder immobilizer while still under anesthesia and are typically discharged home the same day. The operative extremity remains non–weight bearing for the first 6 weeks.

The initial postoperative visit is at 1–2 weeks for wound evaluation and suture removal with initiation of supervised physical therapy. During the first 3 postoperative weeks, motion is limited to the elbow, wrist, and hand, with the shoulder maintained in the shoulder immobilizer. From weeks 3 to 6, controlled passive shoulder range of motion is permitted, with flexion limited to 90°, external rotation to 45°, and extension to 20°. Immobilization is maintained between therapy sessions.

At 6 weeks postoperatively, the shoulder immobilizer is discontinued and active-assisted shoulder motion is initiated. The goal is progression to full, painless shoulder range of motion by approximately 12 weeks. Strengthening is introduced at 12 weeks, emphasizing the rotator cuff, deltoid, scapular stabilizers, biceps, and triceps. At this stage, lower-extremity endurance activities such as running and cycling are permitted.

At approximately 5 months postoperatively, patients begin more aggressive strengthening and plyometric exercises. Initiation of a return to throw program for overhead athletes may commence at this time. Return to full sport participation is typically allowed after 6 months, provided the patient has regained full shoulder range of motion, strength, and sport-specific functional capacity without apprehension or instability symptoms.

## Conclusion

This review article provides a methodical approach to pediatric and adolescent athletes with shoulder instability. Shoulder instability represents a multifactorial condition influenced by skeletal maturity, activity demands, and underlying tissue and bone characteristics. Traumatic anterior instability in adolescents carries a particularly high risk of recurrence, especially in the presence of bone loss, off-track HSLs, and participation in collision sports. Careful individualized risk stratification, attention to physeal anatomy, consideration of athletic goals, and careful selection of stabilization strategies are essential to optimizing outcomes. While arthroscopic stabilization can provide favorable functional outcomes and high return to sport rates, recurrence and revision remain significant concerns in high-risk populations. Ongoing research is continuing to refine treatment algorithms, clarify indications for early operative intervention, and better define long-term functional and degenerative outcomes in the pediatric athlete population.

## Key References

- Cannamela P, Cutler H, Sohn G, Wyatt C, Wilson PL, Ellis HB. Atypical Shoulder Instability Patterns in Adolescents Following Traumatic Anterior Shoulder Dislocation. Am J Sports Med. 2023;51:2018–22. https://doi.org/10.1177/03635465231171129.
  - ○ This was a cross-sectional study of 131 adolescent shoulders (mean age 15.3 years, range 10.5–18.3 years) treated for traumatic anterior shoulder instability at a single institution, in which lesion patterns were analyzed by age younger or older than 15 years. The authors found that atypical soft-tissue lesions were present in 41.8% of patients younger than 15 years compared to only 17.1% in those 15 years and older. Conversely, bony Bankart lesions were more prevalent in the older cohort (34.2% vs. 18.2%), and bone loss was associated with older age at presentation. These findings highlight that injury patterns vary significantly by skeletal maturity, and that treatment must maintain awareness of atypical soft-tissue lesions in younger patients to ensure accurate diagnosis and appropriate surgical management.
- Rossi LA, Tanoira I, Bruchmann MG, Pasqualini I, Ranalletta M. The Latarjet procedure in competitive athletes younger than 20 years old with a significant glenoid bone loss. Shoulder Elb. 2022;14:21–8. https://doi.org/10.1177/1758573220944166 .
  - ○ This was a retrospective cohort study of 60 competitive athletes younger than 20 years old (mean age 16.3 years) with significant glenoid bone loss (> 20%) who underwent the Latarjet procedure with a mean follow-up of 58 months. The study reported that 93% of patients were able to return to sport and 84% returned at the same level as before their injury, with a low recurrence rate of 3.3% and a bone block healing rate of 93%. The total complication rate was notable at 22% with low revision rate of 1.6%. While this remains one of the largest series of adolescent athletes undergoing Laterjet that demonstrates excellent functional outcomes and high return to sport rates, the findings demonstrate low recurrence risk but with increased overall complication profile.
- Kay J, Heyworth BE, Bae DS, Kocher MS, Milewski MD, Kramer DE. Arthroscopic Bankart Repair for Anterior Glenohumeral Instability in 488 Adolescents Between 2000 and 2020: Risk Factors for Subsequent Recurrent Instability Requiring Revision Stabilization. Am J Sports Med. 2024;52:2331–9. https://doi.org/10.1177/03635465241259736.
  - ○ This was a large retrospective cohort study of 488 adolescent patients (mean age 16.9 years, 78% male) who underwent arthroscopic Bankart repair for anterior glenohumeral instability at a single pediatric tertiary care center. Of these patients, 86 (17.6%) required revision stabilization surgery for recurrent instability, yielding cumulative revision rates of 8.8% at 2 years, 16.5% at 5 years, and 20% at 15 years, with revision occurring at a mean of 2.6 years after the index repair. The strongest independent risk factor was the number of preoperative dislocations, with two dislocations conferring over a 7-fold increased risk and three or more dislocations conferring nearly an 11-fold increased risk compared with a single preoperative dislocation. Additional significant risk factors included the presence and size of a Hill-Sachs lesion, younger age, and participation in contact sports. These findings provide clinicians with important prognostic data to help identify which adolescent patients are at highest risk for failure after arthroscopic stabilization and may benefit from earlier consideration of adjunct or alternative procedures.
- Torrance E, Clarke CJ, Monga P, Funk L, Walton MJ. Recurrence After Arthroscopic Labral Repair for Traumatic Anterior Instability in Adolescent Rugby and Contact Athletes. Am J Sports Med. 2018;46:2969–74. https://doi.org/10.1177/0363546518794673.
  - ○ This was a retrospective cohort study of 67 adolescent contact athletes (mean age 16.3 years, 62 of 67 rugby players) who underwent arthroscopic labral repair for traumatic anterior shoulder instability. The overall recurrence rate was 51%, and athletes younger than 16 years had 2.2 times the risk of a further instability episode compared with those 16 years and older, with a recurrence rate of 93% in the youngest cohort. These findings highlight the particularly high failure risk of isolated arthroscopic stabilization in young collision-sport athletes and the importance of age when counseling patients on surgical expectations.

## Data Availability

No datasets were generated or analysed during the current study.
